# Immunotherapy in Hodgkin Lymphoma: Present Status and Future Strategies

**DOI:** 10.3390/cancers11081071

**Published:** 2019-07-29

**Authors:** Theodoros P. Vassilakopoulos, Chrysovalantou Chatzidimitriou, John V. Asimakopoulos, Maria Arapaki, Evangelos Tzoras, Maria K. Angelopoulou, Kostas Konstantopoulos

**Affiliations:** Department of Haematology and Bone Marrow Transplantation, National and Kapodistrian University of Athens, Laikon General Hospital, 11527 Athens, Greece

**Keywords:** Hodgkin lymphoma, brentuximab vedotin, nivolumab, pembrolizumab, relapsed, refractory

## Abstract

Although classical Hodgkin lymphoma (cHL) is usually curable, 20–30% of the patients experience treatment failure and most of them are typically treated with salvage chemotherapy and autologous stem cell transplantation (autoSCT). However, 45–55% of that subset further relapse or progress despite intensive treatment. At the advanced stage of the disease course, recently developed immunotherapeutic approaches have provided very promising results with prolonged remissions or disease stabilization in many patients. Brentuximab vedotin (BV) has been approved for patients with relapsed/refractory cHL (rr-cHL) who have failed autoSCT, as a consolidation after autoSCT in high-risk patients, as well as for patients who are ineligible for autoSCT or multiagent chemotherapy who have failed ≥ two treatment lines. However, except of the consolidation setting, 90–95% of the patients will progress and require further treatment. In this clinical setting, immune checkpoint inhibitors (CPIs) have produced impressive results. Both nivolumab and pembrolizumab have been approved for rr-cHL after autoSCT and BV failure, while pembrolizumab has also been licensed for transplant ineligible patients after BV failure. Other CPIs, sintilimab and tislelizumab, have been successfully tested in China, albeit in less heavily pretreated populations. Recent data suggest that the efficacy of CPIs may be augmented by hypomethylating agents, such as decitabine. As a result of their success in heavily pretreated disease, BV and CPIs are moving to earlier lines of treatment. BV was recently licensed by the FDA for the first-line treatment of stage III/IV Hodgkin lymphoma (HL) in combination with AVD (only stage IV according to the European Medicines Agency (EMA)). CPIs are currently being evaluated in combination with AVD in phase II trials of first-line treatment. The impact of BV and CPIs was also investigated in the setting of second-line salvage therapy. Finally, combinations of targeted therapies are under evaluation. Based on these exciting results, it appears reasonable to predict that an improvement in survival and a potential increase in the cure rates of cHL will soon become evident.

## 1. Introduction

Lymphoid malignancies were among the first to be cured with conventional chemotherapy. Campath-1H, an anti-CD52 monoclonal antibody (moAb), demonstrated significant activity in relapsed/refractory (rr) chronic lymphocytic leukemia in the 1990s [1,2], but immunotherapy revolutionized the treatment of lymphoid tumors with the introduction of the anti-CD20 moAb. rituximab, which was approved for relapsed/refractory low-grade lymphomas in the USA in 1997 and specifically for follicular lymphomas in Europe in 1998 [3]. Rituximab was widely adopted at the beginning of the 21st century, when clear improvements were shown in the outcomes of first-line treatment of a variety of indolent and aggressive B-cell lymphomas [4,5,6,7,8,9,10,11,12,13,14].

This great success, unfortunately, was not applicable to Hodgkin lymphoma (HL) at that time, as rituximab proved useful only in the rare subtype of nodular lymphocyte predominant HL (NLP-HL) [15], although it still remains an off-label option even for this small subgroup. The medical community had to wait until 2011, when improvements in CD30 targeting in the form of brentuximab vedotin (BV) changed the clinical practice with considerable success for heavily pretreated classical HL (cHL) [16] and was used even in the first-line therapy of advanced disease [17]. A few years later, checkpoint inhibitors (CPIs) provided impressive results in subgroups of “hopeless” patients with relapsed/refractory cHL (rr-cHL).

In this review, we will present the current indications of the above immunotherapeutic agents and their potential role in the future. Furthermore, we will review the data on additional molecules and the potential role of the forthcoming chimeric antigen receptor-modified T-cell (CAR T-cell) technology in HL.

## 2. Targeting CD20 in Hodgkin Lymphoma

CD20 is universally expressed in NLP-HL, which represents only 5% of the cases of HL [15]. In cHL, CD20 is expressed in 20–30% of the cases in various percentages of the neoplastic Hodgkin–Reed–Sternberg (HRS) cells and with variable intensity [18]. Given these data, and under the hypothesis that anti-CD20 therapy might modulate the cHL microenvironment, rituximab was tested as a single-agent in a phase II trial with modest activity in rr-cHL. The overall response rate (ORR) was 22% in 22 patients and the median duration of response was 7.8 months, irrespectively of CD20 expression [19]. 

The combination of rituximab with ABVD (R-ABVD) was safe in phase II trials of cHL, resulting in encouraging long-term progression-free survival (PFS) [20,21], but a recent randomized phase II trial of R-ABVD versus ABVD in patients with stage III/IV cHL and an International Prognostic Score (IPS) ≥ 3 was closed prematurely because of the low accrual rate [22]. With only 58 patients randomized instead of 108, R-ABVD was not superior to ABVD. Furthermore, two randomized trials compared the addition of rituximab to BEACOPP-escalated (R-BEACOPPesc) with BEACOPPesc alone in patients with a positive interim positron emission tomography (iPET) after either two cycles of ABVD (Deauville 5-Point Scale (D5PS) score 4–5) [23] or after two cycles of BEACOPPesc (D5PS score 3–5) [24]. In both trials R-BEACOPPesc failed to improve the results of BEACOPPesc alone. Interestingly, however, the toxicity of R-ABVD and R-BEACOPP was not significantly higher than their chemo-only counterparts and, more specifically, there was no additive respiratory toxicity of the combination of rituximab and bleomycin over bleomycin alone [22,24]. Finally, based on the above data, it can be hypothesized that CD20+ cHL cases may have better outcomes than those of CD20− ones under R-ABVD [21,22]. Thus, further testing of R-ABVD versus ABVD in the minority of CD20+ advanced cHL might be warranted, although such a comparison is rather unlikely to be performed in the future as highly active novel agents emerge.

The situation is different in NLP-HL, in which significant clinical activity has been shown with four weekly infusions of single-agent rituximab in phase II trials of rr-NLP-HL, published in 2003 and later. In three such trials, including 43 patients in total, ORR were 94–100% with complete remission (CR) in 53–78% of the patients. The median PFS was as high as 33 months [25,26,27]. Another anti-CD20 monoclonal antibody, ofatumumab, has shown similar clinical activity in rr-NLP-HL. After eight weekly infusions the ORR was 96%, including 61% CRs, and the two-year PFS was 80% in a phase II trial [28]. Notably, in 7/28 patients of this trial, who had been previously exposed to rituximab, ORR and CR rates were similar to the whole series. Rituximab can also produce almost 100% ORR in previously untreated patients with NLP-HL with variable CR rates. However, the results in this setting appear inferior to those of radiotherapy (RT) alone or combined modality therapy for early-stage disease, so that rituximab monotherapy is not recommended as a first-line treatment [25,27,29,30]. In contrast, the combination of rituximab with chemotherapy, even with CHOP (R-CHOP), may be a valid salvage therapy or a first-line option, especially for advanced-stage NLP-HL [31,32,33,34,35].

## 3. Targeting CD30 in Classical Hodgkin Lymphoma

CD30 is the hallmark of cHL and anaplastic large cell lymphoma (ALCL), while it is not expressed in NLP-HL [15]. Serum CD30 levels correlate with tumor burden and have a strong relationship with the outcome of cHL [36]. CD30 offers a reasonable opportunity for targeted therapy, because of its limited expression in normal cells, mainly in small subpopulations of activated B-lymphocytes, T-lymphocytes, and eosinophils. Such targeted therapy was initially attempted in cHL with unconjugated anti-CD30 antibodies. Unfortunately, the clinical activity of those antibodies, namely MDX-060 and SGN-30, was minimal with ORR not exceeding 6% [37,38,39].

The activity of SGN-30 was dramatically increased with the incorporation of monomethyl-auristatin-E (MMAE), a cytotoxic anti-tubulin agent, via an enzyme-cleavable dipeptide linker. The resulting antibody-drug conjugate, brentuximab vedotin (BV) or SGN-35, proved highly active in a phase I trial of heavily pretreated cHL, and the maximal tolerated dose was established at 1.8 mg/kg (up to 100 kg; max 180 mg) administered i.v. every three weeks [40]. The mechanism of action of BV has been described in detail; briefly, involves the attachment of BV on the surface CD30 of the neoplastic cells, BV internalization, the cleavage of the peptide linkers in the lysosomes, and the intracellular release of MMAE resulting in cell cycle arrest and apoptosis [40,41]. Peripheral neuropathy is the most notable and worrisome toxicity of this new agent. 

Later, BV was tested in combination with the standard ABVD as a first-line therapy for advanced-stage cHL, with very promising activity but also prohibitive pulmonary toxicity [42,43]. Thus, the phase I trial established the combination BV-AVD, the addition of BV to a bleomycin-free ABVD, as a candidate for further development in the first-line setting. The dose of BV in this setting was determined at 1.2 mg/kg every two weeks, given concurrently with AVD.

### 3.1. Currently Approved Indications of Brentuximab Vedotin in Classical Hodgkin Lymphoma

The currently approved indications of BV in cHL are summarized in Table 1. They include third or subsequent-lines of therapy, consolidation after autologous stem cell transplantation (autoSCT), and first-line therapy of advanced disease. The basis of these indications is described below.

#### 3.1.1. Third- or Subsequent-Line Therapy

AutoSCT is the standard of care for eligible patients with rr-cHL who achieve CR or partial remission (PR) or even have a stable disease (SD) after second-line salvage chemotherapy. However, roughly only 50% of patients undergoing autoSCT will be cured [44,45]. The expected outcome for those who progress/relapse after autoSCT used to be very poor with a median overall survival (OS) not exceeding 2–3 years [46,47,48,49,50,51,52]. Only a few of these patients can be cured either with allogeneic SCT (alloSCT) or, in a very small minority, with RT to sites of localized disease. In addition to autoSCT failures, third or subsequent-line therapy is also needed in potentially SCT-eligible but clearly chemorefractory patients and those who are ineligible for autoSCT due to advanced age or serious comorbidities.

BV was the first agent to be specifically approved in this clinical setting, initially by the US Food and Drug Administration (FDA) in 2011 and subsequently by the European Medicines Agency (EMA) in 2012 (Table 1, indications 1.1 and 1.2).

##### BrentuximabVedotin in AutoSCT Failures

The approval of BV in patients with cHL who relapse or progress after autoSCT was based on the pivotal phase II trial published by Younes et al. in 2012, where the drug was given i.v. at the dose of 1.8 mg/kg every three weeks until disease progression, prohibitive toxicity, or a maximum of 16 cycles [16]. With the exception of their good performance status (Eastern Cooperative Oncology Group (ECOG) PS 0–1), the 102 patients included in the pivotal trial constituted a highly unfavorable group of rr-cHL, since they had received a median of 3.5 and up to 13 prior regimens, 71% had primary refractory disease and 42% were refractory to the last treatment, 11% had undergone two autoSCTs and the majority had early relapse/progression after autoSCT within a median time of 6.7 months. The conclusions drawn from this trial were briefly the following [53,54]: With PET-based response assessment [55,56], the single-agent activity of BV was the best ever seen at that time with 75% ORR and 34% CRs. In the five-year report, the median PFS reached 9.3 months and the five-year PFS was 22% for all patients. However, the 1/3 of the patients who achieved CR had a five-year PFS of 52% with a possible plateau in the survival curve (although some of them had additional alloSCT) versus <10% for those in PR [53,54]. More importantly, 9/102 patients remained in CR for >5 years without further intervention, thus suggesting that a minority of patients might be cured with one year of BV alone. This was suggested by real-life data from Italy as well [57]. The few CR patients (6/34) who received alloSCT had numerically higher PFS and OS compared with those of the remaining patients, but the number of patients precluded any conclusion regarding the additive value of alloSCT in complete responders to BV (obviously this is not applicable to PRs). Finally, the achievement of metabolic CR to BV, which can be judged after four cycles for the majority of the patients, appears to be the strongest prognostic factor. Other potential prognostic factors for the outcome after BV in the specific setting of autoSCT failure have not been clearly demonstrated. However, real-life data suggest that chemorefractory patients ineligible for autoSCT or those who have received further treatment between autoSCT and BV may have inferior outcomes, as also is the case in patients with B-symptoms or higher tumor burden (bulk) prior to BV [57,58,59]. Such hypotheses need further evaluation.

The data derived by the pivotal study [16] have been generally reproduced in real-life settings with similar ORR, CR, and PFS rates, especially when the inclusion of “worse” patient populations, for example chemorefractory or elderly SCT-naïve patients, is taken into account [57,58,60,61,62,63,64]. These data have been reviewed elsewhere [58]. Similar real-life-based evaluations suggest that the OS of patients with rr-CHL after autoSCT failure has been improved since the introduction of BV [65,66], without this finding having been affected by the application of alloSCT or CPIs [65,66].

In the case of patients with rr-cHL who have responded to BV but have stopped treatment prematurely, BV retreatment is feasible upon further relapse/progression with an ORR of 60% including 30% CRs. These responses lasted for a median of 9.2 months according to the results derived from a small patient cohort [67].

##### Brentuximab Vedotin in Patients Ineligible for AutoSCT

Two subpopulations can be covered by the indication 1.2 (Table 1) of BV, namely failure of ≥2 treatment lines when autoSCT or multi-agent chemotherapy is not a treatment option. The first includes totally chemorefractory patients who fail to achieve even SD with salvage therapy and are considered ineligible for autoSCT. The second includes patients with rr-cHL who have received at least two lines of therapy but are ineligible for autoSCT due to advanced age and/or the presence of significant comorbidities. Data on these clinical scenarios are mainly derived from real-life studies.

Regarding transplant-ineligibility for chemorefractoriness, BV monotherapy at the approved dose of 1.8 mg/kg every three weeks has been studied in several retrospective series, including 9–99 patients each, with the largest being a recently published UK-wide study (Table 2) [57,58,68,69,70,71]. However, these studies did not directly assess the issue of BV efficacy in chemorefractory patients, because an admixture of partial responders (or even CRs in rare cases) was present at percentages ranging from 11% to 38% (Table 2). In addition, a limited admixture with elderly, transplant-ineligible patients was present in some studies [57,68]. Overall, the ORR to BV in this group of transplant-ineligible patients was 40–56% with 25–33% CRs (Table 2), with the only exception of a 53% CR rate in a US study of 15 patients with a more favorable profile in terms of response to salvage therapy pre-BV [70]. With the exception of the latter study, 34–47% of the patients proceeded directly to autoSCT, while in the two larger studies, 60% of the patients actually underwent autoSCT either directly after BV or following additional therapy (Table 2). Notably, some patients underwent autoSCT despite a lack of response to BV, as occasionally done in clinical practice in the absence of other reasonable options. In the UK-wide study, stem cell transplantation (SCT) consolidation, depth of response to BV, ECOG PS 0-1, and absence of anemia at the first relapse predicted a favorable clinical outcome [68]. Collectively, the above data suggest that BV may serve as an effective bridge to autoSCT in a substantial proportion of chemorefractory patients, although this may change in the future if BV is ultimately incorporated into second-line salvage chemotherapy regimens (see below Section 3.2.1.).

Data on the efficacy of BV in case of transplant ineligibility due to age/comorbidity restrictions are scarce because elderly patients are rare in the cHL population. Two recent studies provided a detailed insight into this topic. According to the joint German and British experience, 136 SCT-ineligible patients were treated with BV (85% after ≥2 lines of therapy, mean age 66.7 years) [72]. SCT-ineligibility was usually based on one or more of the following: prohibitive comorbidities (74%), advanced age (57%), patient’s choice (15%), or chemorefractoriness (12%). The concept of SCT-ineligibility was strengthened by the presence of impaired ECOG PS in the majority of the patients (≥ 2 in 61%). The ORR to BV was 74% including 35% CRs, which were very similar to those observed in the pivotal trial after autoSCT failure [16,72]. The median PFS was better, at 15.1 months, albeit without a plateau, making BV a reasonable option for this subgroup of patients. More than 1/3 of the observed deaths were unrelated to HL resulting in a median OS of 17.8 months [72]. The Italian multicenter real-life study included 28 patients >60 years old who had received a median of 2 (1–6) prior regimens, but 39% had also failed autoSCT [57]. The results were similar with an ORR of 68% including 50% CRs.

#### 3.1.2. Consolidation after AutoSCT in High-Risk Patients

Up to 50% of the patients progress or relapse after autoSCT, and the probability of treatment failure can be predicted by several factors [44,45,73,74]. A specific high-risk definition was used in the AETHERA trial to delineate a subgroup of autoSCT recipients who might benefit from further consolidation therapy, including patients with primary refractory disease or early relapse (within one year) and/or extranodal involvement at relapse/progression. In AETHERA, 329 such high-risk patients with rr-cHL who had CR/PR/SD after salvage therapy and had undergone autoSCT were randomized to receive either BV or placebo for 16 infusions every three weeks (~1 year). The primary endpoint of AETHERA was PFS, as judged by an Independent Review Facility (IRF), and the trial was positive, demonstrating a 43% reduction of PFS events [75]. As a crossover for the placebo arm to BV was permitted by the design of AETHERA upon relapse, and this was done in 87% of the placebo failures, there was absolutely no difference in terms of OS, although the planned OS analysis will be performed in 2020. Actually, the three-year OS was >80% in both AETHERA arms being better than expected from historical data. The main outcomes of the AETHERA trial are summarized in Table 3 [75,76].

Interestingly, subgroup analyses demonstrated that BV consolidation is more efficacious in patients with more unfavorable features. Patients with a positive PET prior to autoSCT gained benefit, while PET-negative patients did not, but this analysis was limited by the lack of PET evaluation in 1/3 of the patients and the absence of a predefined definition of PET positivity [75]. When the AETHERA high-risk definition was extended to include additional risk factors (<CR to most recent salvage therapy, B-symptoms at salvage and > 1 salvage regimen required to achieve chemosensitive disease) the benefit of BV consolidation was much more marked in the presence of ≥2 or ≥3 out of the 5 risk factors, but OS again was not affected [75,76]. BV consolidation appears also to be justified in the case where BV is required to enable patients to undergo autoSCT due to chemorefractoriness. Caution should be used, however, to avoid exceeding 16 infusions in total due to the risk of peripheral neuropathy and other toxicities.

#### 3.1.3. First-Line Therapy

##### The BV-AVD Combination

The combination BV-AVD was compared with ABVD in a phase three ECHELON-1 trial [17]. In BV-AVD, BV at a dose of 1.2 mg/kg replaced bleomycin. ECHELON-1 included 1334 adult patients with advanced stage (III/IV) cHL and ECOG PS 0–2, with 64% of them being in stage IV. A new term, the modified PFS (mPFS), was introduced to account for the administration of subsequent anineoplastic therapy due to a positive PET result at the end of treatment. The mPFS events included disease progression/relapse, death of any cause, or modified progression (non-complete response with D5PS score 3–5 according to Independent Review Committee (IRC) assessment but only if followed by subsequent antineoplastic therapy). ECHELON-1 met its primary endpoint demonstrating a 23% reduction in mPFS events per IRC, as reflected by a two-year mPFS rate of 82.1% versus 77.2% for BV-AVD and ABVD, respectively (hazard ratio (HR) 0.77; *p* = 0.03). The benefit was somewhat larger by investigators’ assessment with two-year mPFS-INV rates of 81.0% versus 74.4% (HR 0.73; *p* = 0.007). The two-year OS rate was 96.6% versus 94.9% corresponding to a non-significant 28% reduction in the risk of death (HR 0.72; *p* = 0.19). Thus, FDA approved the first-line use of BV in combination with AVD in advanced stage (III/IV) cHL (Table 1) [17,77,78,79,80]. The results of efficacy and toxicity and the subgroup analysis of ECHELON-1 are summarized in Table 4.

Briefly, as shown in Table 4, febrile neutropenia was more frequent with BV-AVD, being more prominent in elderly patients (≥60 years: 37% versus 17%). This prompted the Independent Data and Safety Monitoring Committee to recommend primary Granulocyte Colony Stimulating Factor (G-CSF) prophylaxis with BV-AVD, which reduced the incidence of febrile neutropenia in the subsequent patients and was established as a recommendation for clinical practice. There were nine deaths during treatment with BV-AVD (7/9 due to febrile neutropenia in patients not receiving primary G-CSF prophylaxis) versus 13 during ABVD (11/13 due to pulmonary toxicity, probably bleomycin-related) for toxic death rates of 1.4% versus 2.0%. Hospitalizations were more frequent with BV-AVD, as were serious adverse events (AEs), but not those leading to any drug discontinuation. Toxicity profiles were different for BV-AVD and ABVD; neutropenia (including febrile neutropenia), peripheral neuropathy, and diarrhea were more common with BV-AVD, while pulmonary toxicity was much more common and potentially lethal with ABVD [17].

Subgroup analysis of the ECHELON-1 data are of particular interest (Table 4). Notably, BV benefit was more marked in males and patients of American origin [17,77]. The most important finding was that patients with more unfavorable characteristics, such as stage IV and multiple extranodal sites, had greater benefit from the incorporation of BV with absolute differences of 7.1–9.1% in two-year mPFS [17]. In addition, a ~4% absolute increase in short-term OS was achieved in the high-risk subgroups with stage IV or any extranodal sites (Table 4). Notably, the survival curves started to separate at 18–22 months, suggesting that the OS benefit was not due to reduction of early mortality [78]. Based on these data derived from preplanned subgroup analyses, EMA approved BV-AVD but only for patients with stage IV cHL [17,79].

An interesting subanalysis of ECHELON-1 focused on the value of iPET [80]. Surprisingly, the occurrence of a positive iPET (D5PS score 4–5) was <10% in both arms, in other words, much less frequent than the 16–24% expected with ABVD for a population with advanced cHL [81]. After ABVD, the 3-year PFS was 79.5% versus 51.5% for iPET-negative and iPET-positive patients, respectively, although treatment escalation was minimal, pointing to a higher false positive rate in ECHELON-1. The negative predictive value (NPV) fell into the expected range for stage III/IV [81,82,83,84]. However, following BV-AVD, the 3-year PFS was 85.8% versus 67.7%, again with minimal treatment escalation in response to a positive iPET. This suggests, if validated, that BV-AVD might be associated with improved NPV of iPET and an acceptable chance of curing despite iPET positivity, both of which are not the case with ABVD.

##### The Role of Brentuximab Vedotin in Elderly Patients

A prespecified analysis of ECHELON-1 according to age demonstrated that BV-AVD was equally effective as ABVD in elderly patients ≥60 years old. A detailed analysis of the elderly ECHELON-1 subgroup (186/1334 patients; 14%) was presented at the 2018 American Society of Hematology (ASH) meeting [85]. The main results are shown in Table 4. Elderly patients had exactly the same mPFS with BV-AVD (*n* = 84) versus ABVD (*n* = 102) (HR = 1.00; *p* = 0.99). When only stage IV patients were considered (51 and 67, respectively), the difference was still non-significant, but the two-year mPFS rates were 71.3% versus 66.1% (HR = 0.80; *p* = 0.51). The difference was larger, but still non-significant, if investigator-assessed PFS was considered (Table 4). Interestingly, toxic deaths with BV-AVD were only 2/84 (surprisingly none of febrile neutropenia) versus 5/102 with ABVD (four from respiratory complications). Although ECHELON-1 was not powered to demonstrate a difference in elderly patients, BV-AVD could be an attractive option for two reasons: First, it allows the complete omission of bleomycin in a population particularly vulnerable to bleomycin lung toxicity, which in addition is more likely to be lethal. Second, if bleomycin were omitted after ABVD × 2 according to RATHL [82], this would be applicable roughly in 80% of the patients; however, 20% would remain iPET-positive and would have a poor expected outcome, but they could not undergo treatment escalation in the form of BEACOPP-escalated. In contrast, the expected outcome after a positive iPET following BV-AVD × 2 is not as disappointing as discussed above [80]. Another schedule of sequential BV-AVD-BV was proposed by Evens et al., with promising results [86], while results similar or inferior to ECHELON-1 were reported by the German Hodgkin Study Group (GHSG) with the B-CAP (Brentuximab vedotin, cyclophosphamide, adriamycin, prednisone) regimen, corresponding to a BV-CHOP without vincristine [87] (Table 5).

In elderly patients who were not good candidates for ABVD, 12 cycles of BV-dacarbazine provided 100% ORR with 62% CRs, but responses were rather short-lived with a median PFS of 17.9 months (~49% at two years) [88]. The BV-bendamustine combination was too toxic, with 10% toxic deaths in this vulnerable population of even higher age, and was not recommended (Table 5) [88]. In contrast, BV-bendamustine was not as toxic in the HALO trial, where patients were not negatively selected, but the final PFS results are still awaited [89].

Finally, BV monotherapy was evaluated in two studies of advanced-stage elderly patients considered unfit for standard chemotherapy [90,91] or with cardiorespiratory compromise at any age [91]. The ORR were 84–92% with variable CR rates (26% and 73%, respectively); however, most responses were short-lived with median PFS of 10.5 and 7.4 months, respectively (Table 5).

Generally, BV monotherapy and combinations with non-anthracycline regimens appear to be inferior to BV-AVD and its variants in elderly patients. In patients ineligible for ABVD or BV-AVD, these approaches can provide reasonable alternatives, although their value compared with older chemotherapeutic approaches such as MOPP (mechlorethamine, vincristine, procarbazine, predinisone), ChlVPP (chlorambucil, vinblastine, procarbazine, predinisone), or other regimens is uncertain.

### 3.2. Brentuximab Vedotin in Clinical Settings Outside the Approved Indications

#### 3.2.1. Incorporation into Second-Line Salvage Regimens

It is peculiar that BV gained third-line and post-autoSCT consolidation and then the first-line indication without being tested in the second-line salvage setting with an intention to receive regulatory approval. However, BV has been successfully incorporated in the second-line salvage therapy in combination with platinum-based regimens or bendamustine or even with nivolumab in patients who are candidates for autoSCT. The sequential administration of BV and augmented ICE or other regimens, the incorporation of BV into BrESHAP, BV-DHAP (BrAVE trial) and BV-ICE, and the combination of BV with bendamustine or nivolumab in the SCT setting are summarized in Table 6 [92,93,94,95,96,97,98,99,100,101,102].

The overall impression from these studies is that BV-salvage combinations, either sequential or concurrent, can improve the response rates of conventional salvage regimens with metabolic CRs in 70–80% of the patients, allowing very high SCT rates of 85–100% and finally resulting in two-year PFS rates in excess of 70%. However, the metabolic CR rates should be compared with caution with the conventional responses achieved by the traditional salvage regimens, while it should be kept in mind that BV–chemo has not been directly compared with conventional salvage strategies. With the additional limitations of the moderate number of patients included in these studies and their higher toxicity, BV–chemo combinations appear to be very promising in increasing the number of patients who will be cured with the second effort, including autoSCT.

Two additional observations are worth mentioning from the above studies: First, BV monotherapy can induce metabolic CR in 25–35% of patients in the second-line and allow a usually curative SCT without exposure to salvage chemotherapy [92,93,94,95,96]. Second, BV-bendamustine or BV-nivolumab might potentially replace platinum- or gemcitabine-based chemotherapy before autoSCT as an alternative, less (or equally) toxic, outpatient regimen [100,101,102].

#### 3.2.2. Other First-Line Applications

##### Advanced Stages: Incorporation into a Modified BEACOPP-Escalated Backbone

The improved results of BV-AVD appear inferior to those of six BEACOPP-escalated cycles [103,104,105] or BEACOPP-based response-adapted strategies [24,106,107], in which the three-year PFS exceeded 90%. In an effort to reduce the toxicity of BEACOPP-escalated, the German Hodgkin Study Group (GHSG) incorporated BV into the backbone of the regimen, omitting bleomycin and vincristine due to concerns of additive pulmonary and neurologic toxicity. The resulting regimen was BrECAPP. Furthermore, a four-day dexamethasone course replaced the 14-day prednisone one in order to avoid long-lasting steroid treatment and particularly the exposure to steroids during neutropenia, and dacarbazine replaced procarbazine for reasons of fertility preservation and leukemogenicity, thus generating the BrECADD regimen. BrECAPP and BrECADD were compared in a randomized phase two study and BrECADD was judged to be similarly effective but less toxic [108]. BrECADD is now being compared with six cycles of BEACOPP-escalated in the HD21 trial of the GHSG (NCT02661503).

##### Localized Stages

BV has been also evaluated in concurrent or sequential combination with AVD as a first-line therapy in localized HL in three multicenter phase II studies. The goal was to omit RT in non-bulky cases [109,110] and increase efficacy in combination with RT in predominantly bulky unfavorable cases [111]. The preliminary results on a series of 30–40 patients were promising and are summarized in Table 7. However, the number of patients is small and the follow-up time is too short to draw conclusions.

#### 3.2.3. BV Combinations with Other Novel Agents

BV has been recently tested in combination with nivolumab and ipilimumab in a phase I trial of 22 patients who had received a median of two prior regimens (range 1–5, 9 after prior SCT, only 1 after prior BV). The results appeared very promising, although with more grade three adverse events than observed with doublets of BV plus nivolumab or BV plus ipilimumab [112]. The preliminary results of a phase II trial of BV in combination with ibrutinib have also been presented [113].

## 4. Targeting Immune Checkpoint Pathways in Classical Hodgkin Lymphoma

### 4.1. Rational for Checkpoint Inhibitor Therapy in Classical Hodgkin lymphoma

The outcome of patients who fail both autoSCT and BV is extremely poor. Conventional chemotherapy or experimental approaches based on small molecules provide disappointing PFS rates of only 3.5 months median duration and a median OS of ~2 years [114]. AlloSCT can be curative in this setting, but several limitations make it applicable only in a minority of patients. Thus, novel approaches are urgently required. Restoring the immune response against the HRS cells rather than targeting them with cytotoxic agents appears to be the way to go.

The ability of the neoplastic cells to escape from immune surveillance has been recently considered as a hallmark of cancer [115]. Cancer cells frequently exploit immune checkpoints, such as Programmed Death-1 (PD1) and PD1 Ligand (PDL1), to cause exhaustion of tumor infiltrating lymphocytes (TILs). Physiologically, immune checkpoints regulate inflammation and immune response by preventing the overactivation of T-cells, thus providing protection from hypersensitivity reactions and autoimmunity. PD1 is expressed by activated T-cells, NK cells, T-regulatory cells (T-regs), T-follicular helper cells (T-FH), B cells, and macrophages. The ligands PDL1 and PDL2 are expressed on the surface of tumor cells and tumor associated macrophages (TAMs). PD1–PDL1/PDL2 interactions cause crosstalk with (HLA)-T-cell receptors (TCR) resulting in inactivation of PI3K/AKT and RAS-MEK pathways and thus leading to cell cycle arrest, changes in metabolism, and a decrease in T-cell cytokine production [116]. 

Hodgkin lymphoma is perhaps the most “inflamed” human tumor, as HRS cells account for a small minority of < 1–10% of the tumoral mass. The HRS cells use various mechanisms in order to immune-escape, including downregulation of surface HLA class I and II expression, mainly through β_2_-microglobulin mutations/deletions and CIITA mutations, respectively, as well as secretion of paracrine factors, such as IL-10 and TGF-β, which attract T-regs and myeloid-derived suppressor cells and lead to exhaustion of CD8+ T-cells [116]. The upregulation of the PD1-PDL1/2 axis is a major immune escape pathway in cHL because of the abundance of PDL1 and PDL2 protein expression in the overwhelming majority of cases. This is because 97% of cHL cases harbor alterations of the 9p24.1 locus, which includes *PDL1, PDL2*, and *JAK2* genes [117], consisting of copy gains (56%), amplification (36%), and polysomy (5%) by FISH analysis. Apart from HRS cells, PDL1 expression is amplified, as it is also expressed by surrounding TAMs. The disruption of the PD1–PDL1/2 interaction by monoclonal antibodies called immune checkpoint inhibitors is an attractive treatment strategy, which, in fact, revolutionized the treatment of cHL over the last five years.

The disruption of the PD1–PDL1/2 interaction results in the reactivation of cytotoxic CD8+ T-cells against HRS cells. However, CD4+ T-cells represent the majority of reactive cells in the cHL microenvironment [118]. Recently, it was shown that rosettes of CD4+/PD1+ T-cells [119] are formed around HRS cells, mimicking the antigen presenting cell (APC)-T-cell immunological synapse [118]. Although Th2 and Treg phenotypes contribute to the cHL immunosuppressive microenvironment [118], there is also evidence that CD4+ T-cells can exhibit antineoplastic activity [118,120]. Finally, it was recently reported that in HL, NK cells are also exhausted by the PD-1/PDL-1 axis and this is reversible with immune checkpoint inhibitors [121]. Given the HLA class I loss and the limited number of CD8+ T-cells in the microenvironment, it is likely that the main mechanism of the PD1 blockade in cHL is not primarily mediated by CD8+ cytotoxic T-cells. The exact mechanism of action remains unknown, but the above studies could indicate that it might be CD4− or NK-mediated. However, more research is needed.

Nivolumab and pembrolizumab, human and humanized IgG4 monoclonal antibodies, respectively, are the first checkpoint inhibitors used in cHL, targeting PD-1. The PD-1 inhibitors sintilimab and tislelizumab were subsequently developed in China. An older checkpoint inhibitor used in melanoma, ipilimumab, was tested in combination with BV and/or nivolumab in rr-cHL, as previously discussed [112].

### 4.2. Results of Nivolumab and Pembrolizumab in Approved Indications of Relapsed/Refractory Classical Hodgkin Lymphoma 

Following the outstanding results obtained in heavily pretreated cHL in phase I trials with acceptable toxicity [122,123], nivolumab and pembrolizumab were further developed in the Checkmate 205 and KEYNOTE-087 phase II trials, which enrolled 243 and 210 patients respectively and now have relatively mature results available [124,125,126,127,128]. Both trials included three arms (cohorts) of patients with rr-cHL, each one reflecting a different clinical scenario in terms of previous autoSCT and BV exposure. The Arm B of Checkmate 205 and the cohort 1 of KEYNOTE-087 described the same clinical scenario of rr-cHL after both autoSCT and BV failure. While all Checkmate 205 arms required a previous autoSCT, the 2nd cohort of KEYNOTE-087 included patients ineligible for autoSCT mainly due to chemorefractoriness but also due to age/comorbidity restrictions. This information forms the basis for the different approved indications of the two agents (Table 1). Both trials included heavily pretreated patients, as described in detail in Table 8, in comparison to the patients’ characteristics in the sintilimab and tislelizumab trials. The overall results of these trials are also described in Table 8. The description of the arms/cohorts and overall results of both trials as well as the results according to arm/cohort are presented in Table 9. It is important to note that elderly patients were underrepresented in the PD1-inhibitor trials due to the requirement of a previous autoSCT (<10% of study population) with the exception of cohort 2 of KEYNOTE-087 where 18% of 81 SCT-ineligible patients were ≥ 65 years old. Furthermore, recruitment was restricted to patients with ECOG PS 0–1 in both trials. Consequently, experience with PD-1 inhibitors in elderly patients and those with impaired PS is limited.

Drug dosing and duration of treatment are given in Table 8. The dose of nivolumab in Checkmate 205 was 3 mg/kg every two weeks until disease progression or unacceptable toxicity. This was later modified in clinical practice to a 240 mg fixed dose. Especially in arm C, nivolumab could be stopped in case of a sustained CR for 1 year and could be resumed at relapse, if this occurred within two years from the last dose [124]. In KEYNOTE-087, pembrolizumab was given by i.v. at the currently approved fixed dose of 200 mg every three weeks [125]. This is much lower than the dose of 10 mg/kg every two weeks used in the phase one KEYNOTE-013 study [123]. Pembrolizumab was given until progression or intolerable toxicity or investigator decision or a maximum of two years. However, in all three cohorts, pembrolizumab could be discontinued in case of CR, if the patient had received ≥ 2 doses after CR documentation and had completed ≥6 months of treatment. Later on, the recommended dose of pembrolizumab as monotherapy in clinical practice was modified to include not only 200 mg every three weeks but also 400 mg every six weeks.

A close look at Table 8 and Table 9 permits the following general conclusions to be drawn from Checkmate 205 and KEYNOTE-087: First, based on independent central review, the ORR for both agents was 69% with CRs at 16% and 22% for nivolumab and pembrolizumab, respectively. These rates were better for nivolumab according to investigators’ assessment, while, as expected, the best OR and CR rates with pembrolizumab were also higher. Second, the ORRs were similar across the arms/cohorts of both trials, but the CR rate appeared higher in the less heavily pretreated Arm A of Checkmate 205. Third, the median PFS was also highest in Checkmate 205 Arm A and lowest in the most chemorefractory group, namely cohort 2 of KEYNOTE-087. However, such comparisons are only indicative, since the studies were not designed to compare the individual arms (cohorts). Fourth, the two-year OS rate exceeded 85% in all arms of the nivolumab trial and was 89.4–92.5% in the three pembrolizumab cohorts. Finally, the Lugano 2014 response criteria [129] resulted in higher CR rates compared to the Cheson 2007 [55] ones within the KEYNOTE-087 [130].

Similar outcomes to those seen in clinical trials have been reported in real-life settings as well [131,132].

### 4.3. Clinical Results of Other Checkpoint Inhibitors in Relapsed/Refractory Classical Hodgkin Lymphoma

Sintilimab and tislelizumab are PD-1 inhibitors developed and evaluated in China. In contrast to nivolumab and pembrolizumab, <20% of the patients were exposed to autoSCT and even fewer to BV in the phase II trials of these agents, because autoSCT is not affordable to many Chinese patients and BV is not approved in China. Sintilimab and tislelizumab were evaluated exclusively in Chinese Centers in two phase II trials of patients with rr-cHL who had failed ≥2 prior therapies, namely the ORIENT-1 (*n* = 92) [133] and BGB-A317-203 (*n* = 70) [134], respectively. Obviously, these populations had been exposed to lesser amounts of prior therapy compared to the populations of Checkmate 205 and KEYNOTE-087. Thus, the results of sintilimab and tislelizumab, as described in Table 8, are not directly comparable to those of nivolumab and pembrolizumab. Furthermore, the follow-up time is still short. Overall response rates appear numerically higher and PFS and toxicities similar to nivolumab and pembrolizumab, but they were derived in clearly more favorable populations of patients with rr-cHL. Sintilimab has been approved in China and will be evaluated in the western world. 

In conclusion, the Checkmate 205 and KEYNOTE-087 phase II trials confirmed the high response rates, which were almost equally applicable in the pre-specified different clinical circumstances of rr-cHL in terms of BV and ASCT pretreatment, as well as in the well-tolerated side effects of checkpoint inhibitors. Furthermore, the mid-term results confirm that responses can be durable. After appropriate testing, sintilimab and tislelizumab might provide additional options, especially in the context of the rising costs of rr-cHL treatment, which are becoming increasingly significant.

### 4.4. Topics of Special Interest on Checkpoint Inhibitor Therapy

Obviously, due to their unique mechanism of action involving the “motivation” of the immune system against the HRS cells, checkpoint inhibitors’ clinical benefit is based not only on inducing CRs but also on maintaining a prolonged “equilibrium” in partial or non-responders or even in patients with PD according to conventional definitions. In this peculiar clinical setting, different drug-specific prognostic and predictive factors may be applicable. The “immunologic” mechanism of action is also linked to rather rare but unique “immunologic toxicities” and may interfere with subsequent or previous alloSCT, modulating the toxicity profile of the procedure.

#### 4.4.1. Criteria of Response and Treatment Beyond Progression 

Treatment with some novel agents and particularly checkpoint inhibitors, may induce an early immune-mediated tumor flare [135,136]. This “pseudoprogression” may not be appropriately recognized, thus resulting to treatment discontinuation. The Lymphoma Response to Immunomodulatory therapy Criteria (LYRIC) criteria of response were developed in response to this observation in order to prevent inappropriate drug discontinuation [136].

An early amendment of the Checkmate 205 permitted patients to continue nivolumab after the occurrence of investigator-assessed disease progression, if pre-specified criteria were met. These included stable performance status and perceived clinical benefit per investigator assessment. However, treatment was withdrawn in the case of further progression defined by ≥10% further increase in tumor burden. Among 130 patients who developed PD, 80 (62%) were treated beyond progression (TBP) and 50 were not. The one-year OS rate after progression for those TBP was 84% and was significantly better than the 61% rate in patients not eligible to be TBP [124,137]. Interestingly, at the last report, the three-year OS of Checkmate 205 patients who achieved a CR was clearly >90%, while it was ~80% for both PRs and patients with SD as the best response, despite their clearly different PFS. However, even the minority of patients (11%) who had PD as the best response, had a three-year OS of 50% or more [128]. These data suggest that checkpoint inhibitors exert a prolonged beneficial effect on the disease, which is not solely determined by the depth of response, and highlight the importance of TBP, as long as a clinical benefit is being obtained.

#### 4.4.2. Potential Prognostic Factors

Roemer et al. showed that 9p24.1 amplification is more frequent in advanced cHL and constitutes an independent, adverse prognostic factor for the outcome of first-line therapy [117]. 

In a retrospective study of 30 patients with rr-cHL treated with nivolumab after autoSCT and mostly after BV, white blood cell (WBC) count and relative eosinophil count (REC%) were independent prognostic factors for PFS with hazard ratios of 5.15 and 0.14, respectively. Patients with both high WBC and low REC% had a median PFS of 110 days, while those with only one adverse factor had a median PFS of 350 days [138]. At a biological level, high PDL1 expression from an underlying amplification or high-level copy gain and surface HLA class II expression predicted for a favorable outcome in patients treated with nivolumab within the Checkmate 205 trial [139]. Although most patients responded to nivolumab, those with low-level 9p24.1 alterations and subsequently lower PDL1 expression had shorter PFS. Surprisingly, 92% of CRs had no surface HLA class I expression, while 67% retained surface HLA class II expression and 25% had decreased surface HLA class II expression. However, extensive prognostic studies are still lacking.

#### 4.4.3. Toxicities of Checkpoint Inhibitors

The AE profile of checkpoint inhibitors is different from that of chemotherapy, with immune-related adverse events (IMRAEs) being their most notable toxicity, which occurs at a median time of 12 weeks (0–62) after treatment institution. The side effects are usually mild and easily manageable. No drug-related deaths were observed in the Checkmate 205 and KEYNOTE-087 trials. The most frequent drug-related AEs observed in these trials are described in Table 8. Treatment discontinuation rates due to drug-related AEs were rather high in both trials (7–11% of the patients), with the observed events consisting of pneumonitis, autoimmune hepatitis, and infusion-related reactions, as also described in Table 8. However, peculiar toxicities, mainly IMRAEs can be observed and can be the cause of treatment discontinuation in isolated patients (myocarditis, myelitis, myositis, epilepsy, organizing pneumonia, cytokine release syndrome, etc.). IMRAEs include the above as well as other rare complications. In this context, thyroid disorders (hypothyroidism, thyroiditis, and rarely hyperthyroidism), rash, hepatitis, pneumonitis, colitis, diabetes, hypophysitis, adrenal insufficiency, autoimmune nephritis, and other manifestations should be considered in the case of clinical suspicion or patients should be carefully monitored over time for the appearance of some of these complications. The unique AE profile of checkpoint inhibitors has led to the publication of guidelines devoted to their recognition, follow-up, and management [140]. 

#### 4.4.4. Checkpoint Inhibitors and AlloSCT

The introduction of PD-1 inhibitors in the treatment of rr-cHL after autoSCT has challenged the role of alloSCT in the clinical setting [141]. There is some evidence from preclinical studies that immune activation resulting from checkpoint blockade might be associated both with increased immune-related toxicity and antineoplastic activity [142]. Although the overall incidence of acute graft versus host disease (aGvHD) in allogeneic transplant recipients following PD-1 inhibitor therapy appears similar to what is expected, PD-1 inhibitors before alloSCT may be associated with more severe (grade 4) aGvHD, hyperacute GvHD, more frequent liver venoocclusive disease, and the occurrence of a non-infectious febrile syndrome that responds to steroids in a sizeable minority of patients, as shown in Table 10 [124,142]. Despite these complications, treatment-related mortality (TRM) does not appear to appreciably increase. Due to the potential increase of early post-alloSCT toxicity, FDA included a warning and precaution label for alloSCT complications in the prescribing information of nivolumab. Furthermore, recent evidence suggests that despite some risk of alloSCT complications, the efficacy of alloSCT may be actually increased by prior exposure to PD-1 inhibitors. Thus, prior anti-PD-1 treatment should not preclude a subsequent alloSCT [124]. Although, a six-week anti-PD-1 treatment-free period is recommended before alloSCT, published studies (on rather small patient populations) have not shown a clear association between nivolumab plasma levels at the time of SCT or the time from the last anti-PD-1 dose and alloSCT and the incidence of severe aGvHD or TRM [124,142].

Finally, although PD-1 inhibitors are very active and produce durable responses when administered after alloSCT, their immune effects are even more pronounced in this setting, including severe and steroid-refractory, early aGvHD [143,144]. These complications could be ameliorated by avoidance of PD-1 inhibitors for six months after alloSCT and treatment at lower doses (e.g., 0.5 mg/kg nivolumab).

### 4.5. Clinical Results of Checkpoint Inhibitors Outside the Approved Indications

#### 4.5.1. Combinations that May Increase Efficacy of PD-1 Inhibitors

In 2016, Falchi et al. published their experience on 10 patients, suggesting that treatment with nivolumab or pembrolizumab might result in higher CR rates, as observed in 5/5 patients who had been previously exposed to 5-azacitidine in the context of a phase one trial [145]. The authors hypothesized that hypomethylating agents might have an immune priming effect and potentiate the efficacy of PD-1 inhibitors. Indeed, it has been shown that decitabine can boost T-cell function.

This hypothesis was tested in a very recent single-center, two-arm, open-label phase II trial conducted in the Chinese People’s Liberation Army General Hospital [146]. The trial included 86 patients with rr-cHL with ECOG PS 0–1 after failure of ≥2 previous lines of therapy. In fact, they had failed a median of four lines of therapy, 26% had failed autoSCT, 8% had failed BV and 25/86 had been previously exposed to PD-1 inhibitors, mainly nivolumab. The PD-1 inhibitor used in this trial was camrelizumab (SHR1210), a fully humanized IgG4/κ antibody with previously shown activity against nasopharyngeal and esophageal carcinoma. The 25 patients who had previously received PD-1 inhibitors, were treated with a combination of decitabine 10 mg/day i.v. on days 1–5 and camrelizumab 200 mg i.v. on day 8 every 3 weeks. The 61 PD-1 inhibitor-naïve patients were randomized at a ratio 1:2 to receive either camrelizumab 200 mg every 3 weeks (*n* = 19) or the combination (*n* = 42). Treatment was planned until PD or unacceptable toxicity with the option of discontinuation in the case of sustained CR for >1 year.

In patients with prior PD-1 inhibitor exposure, the ORR to the combination was 52%, being higher in those with acquired compared to primary resistance (62% vs. 42%). The CR rate was 28%. At one year, PFS and duration of response rates were 59% and 81%, respectively.

In PD-1 inhibitor-naïve patients, the ORR was similar for the combination versus camrelizumab monotherapy (95% versus 90%) but the CR rate, assessed by PET/CT criteria, was significantly higher with the combination (71% versus 32%, *p* = 0.003). Among SCT failures, the CR rates were 80% versus 20% (*p* = 0.089). The one-year PFS was 89% versus 59% for the combination and camrelizumab monotherapy, respectively, and the proportion of durable responses at one year were 89% versus 60%.

Cherry hemangiomas were the most frequent AEs occurring in 84–87% of patients in both arms, but they were mild and self-limited. Leukopenia was much more frequent in the combination arm (76% versus 32%) with grade 3/4 leukopenia being observed only in the combination arm (37% versus 0%). The frequency of IMRAEs was similar in the two arms leading to treatment discontinuation in two patients of the combination therapy arm.

Although long-term data are still pending, if validated for other PD-1 inhibitors, the combination with hypomethylating agents appears to be an extremely promising, exciting, and novel approach.

#### 4.5.2. PD1-Ligand-1 (PDL-1) Inhibitors in Classical Hodgkin Lymphoma

Avelumab is an IgG1 fully human monoclonal antibody binding selectively to PDL-1, thus leaving intact the PD-1/PDL-2 axis. In the phase 1 JAVELIN trial, avelumab was given in five different doses/schedules to 31 patients with rr-cHL; 5 after autoSCT, 8 after alloSCT, and 18 SCT-ineligible. The ORR was 55% with 7% CRs with responses seen across all dosing schedules (ORR ranging from 14% to 83%). Toxicities were acceptable. Thus, avelumab is another clinically active checkpoint inhibitor, which could be further tested in rr-cHL [147].

#### 4.5.3. Consolidation with PD-1 Inhibitors after AutoSCT

Similar to the AETHERA design, consolidation pembrolizumab was evaluated in a phase II trial after autoSCT in 30 patients with high-risk rr-cHL. Treatment was initiated within 21 days from discharge after autoSCT performed following achievement of metabolic CR (90%) or PR (10%) after 2–3 lines of therapy. Pembrolizumab was administered at a dose of 200 mg i.v. every three weeks for up to eight cycles. Most patients had high-risk features: 87% would have been eligible for AETHERA and 26% had ≥2 risk factors. At 19 months (~1.5 years) PFS and OS were 81% and 100%, respectively, with similarly good outcomes for high-risk patients or those with multiple risk factors. There were no unexpected toxicities. These results compare favorably with AETHERA and historical data, but should be interpreted with caution because of the small number of patients and use of different eligibility criteria [148].

#### 4.5.4. Incorporation of PD-1 Inhibitors into First-Line Regimens

Both nivolumab and pembrolizumab have been preliminarily evaluated in the frontline therapy of advanced cHL in phase II trials [149,150]. Given that both agents can cause pneumonitis, they were combined with AVD rather than ABVD, omitting bleomycin due to concerns of additive lung toxicity. Both trials included a brief course of four nivolumab or three pembrolizumab infusions prior to proceeding with the nivolumab-AVD combination or AVD alone in order to assess the efficacy of monotherapy. 

In the Checkmate 205 Arm D, 51 patients with stage III/IV or IIB-bulky and/or extranodal cHL were treated with nivolumab × 4 followed by nivolumab-AVD × 6. The primary endpoint was safety and tolerability, as evaluated by the occurrence of TRAEs grade 3–5. A single 68 year old patient died 38 days after the last dose due to febrile neutropenia, respiratory infection, and congestive heart failure. There were no cases of pneumonitis. The ORR to nivolumab monotherapy was 69% including 18% CRs. The nine-month mPFS was 92% [150].

In another trial, 15 patients with cHL (8 early unfavorable and 7 advanced) received three standard pembrolizumab cycles prior to proceeding with AVD. Among 13 evaluable patients, 6 achieved a D5PS score of 2–3 with pembrolizumab monotherapy while PET normalized after AVD × 2 in all 12 eligible patients. The results still remain very preliminary but promising [149].

## 5. Other Immunotherapy Options in Hodgkin Lymphoma

The better understanding of the biology of HL has led to the discovery of novel therapeutic targets. Selected targets for potential immunotherapy of HL, their function, and relevant clinical trials are summarized below.

### 5.1. Monoclonal Antibodies and Immunoconjugates

ADCT-301 (Camidanlumab Tesirine; Cami-T) is a novel antibody–drug conjugate composed of a human monoclonal antibody against CD25 and a potent pyrrolobenzodiazepine dimer (PBD) toxin, a DNA-crosslinking agent. CD25, the α-chain of the interleukin-2 (IL-2) receptor, is expressed on the cell surface of several lymphomas, including HL. Cami-T was tested in a phase I, open-label, dose-escalation and dose-expansion multicenter study in 60 patients with rr-cHL. Patients had received a median of five (range: 2–15) prior lines of therapy. The drug was given i.v. every three weeks for a median of three cycles (range: 1–15) at dose levels 5–300 μg/kg. Although the maximal tolerated dose was not reached, the recommended dose for expansion was set at 45 μg/kg. Treatment was discontinued because of AEs in 28% of the patients. In the 26 evaluable patients of the 45 μg/kg cohort, the ORR was 81% with 50% CRs and this was independent from prior exposure to autoSCT, BV, and PD-1 inhibitors. The median duration of response was 7.7 months. The side effect profile included peripheral edema and/or effusions, a potentially class-specific side effect of pyrrolobenzodiazepines, in 27% of the patients, fatigue, maculopapular rash, and liver function test abnormalities. Three patients developed IMAEs including two cases of Guillain–Barre and a case of thyroiditis [151].

AFM13, a bispecific anti-CD30/CD16A monoclonal antibody construct, exerts its antitumor effect through CD16A-mediated activation of natural killer cells. AFM13 has been evaluated in a phase one dose-escalation clinical trial of 28 patients with rr-cHL. At doses of 0.01–7 mg/kg, 11.5% of patients achieved PR and 50% SD for an overall disease control rate of 61.5%. At doses ≥1.5 mg/kg the ORR was 23% and the disease control rate 77% [152].

Several other potential therapeutic targets have been tested in clinical trials with poor to modest results. Galiximab, a primatized IgG1 monoclonal antibody against CD80 that is aberrantly expressed by the HRS cells, demonstrated disappointing activity in a phase II clinical trial of rr-HL [153]. Lucatumumab (HCD122), a monoclonal antibody targeting CD40, demonstrated low-to-moderate activity in a phase II trial of 18 rr-HL patients [154]. Other agents that have been investigated but are not currently under clinical development due to poor patient accrual, significant toxicity, and/or disappointing results, include antibodies against TNF-related apoptosis-inducing ligand (TRAIL)(AMG655) and CD25 (RFT5-SPMT-dgA). A phase II trial of alemtuzumab (Campath-1H) in rr-HL was terminated due to slow accrual (ClinicalTrials.gov identifier: NCT00129753). Alemtuzumab is currently being tested in a phase II trial in combination with the dose-adjusted-EPOCH regimen (ClinicalTrials.gov identifier: NCT01030900).

TNX-650, a monoclonal antibody targeting IL-13, is currently under investigation in a phase I/II study of rr-HL, but no data have been published yet (ClinicalTrials.gov identifier: NCT00441818). Finally, the lymphocyte activation gene-3 (LAG-3) is an immune checkpoint molecule, expressed in the cHL microenvironment. Through the interaction with its ligand, MHC class II, LAG-3 suppresses T-cell function and mediates T-cell exhaustion in combination with PD-1 [155]. BMS-986016, a monoclonal anti-LAG-3 antibody, is currently being evaluated in a phase two/three trial in rr-HL, alone or in combination with nivolumab (ClinicalTrials.gov Identifier: NCT02061761).

### 5.2. CAR T Cells

The application of chimeric antigen receptor-modified T cell (CAR-T cell) therapy is currently restricted to aggressive B-cell lymphomas and B-cell acute lymphoblastic leukemia. CD30, CD123, and Epstein-Barr virus (EBV)-related proteins can provide targets for CAR-T cell approaches in cHL, which are in an early stage of development with still uncertain but promising results [156,157,158,159,160]. 

Ramos et al. reported the administration of autologous CD30.CAR-Ts in seven patients with rr-HL with promising results and no toxicity. Of seven patients, 1 achieved complete response (CR) with a duration of more than 2.5 years after the second infusion of CD30.CAR-Ts, 1 maintained a CR for almost 2 years, and 3 had transient stable disease [157]. The same investigators performed a phase one study (RELY-30) in which nine patients with rr-HL received CD30.CAR-Ts at two dose levels (2 × 10^7^ and 1 × 10^8^ cells/m^2^) after lymphodepleting chemotherapy with cyclophosphamide 500 mg/m^2^ and fludarabine 30 mg/m^2^ daily for three days, trying to exploit a potential boosting effect on expansion and eventually on efficacy of these CAR-Ts. Maximum toxicity consisted of the appearance of grade 1 cytokine release syndrome in four of the patients. At 6 weeks after infusion, 6/8 evaluable patients achieved a CR of 6 weeks to 6 months duration, while 2 had PD [158]. 

Grover et al. presented impressive results in another phase Ib/II study of CD30.CAR-Ts infused after lymphodepletion in 18 patients with rr-CD30+ lymphomas (16 HL). Two dose levels were examined at the phase Ib portion of the trial: 1 × 10^8^ CAR-Ts/m^2^ and 2 × 10^8^ CAR-Ts/m^2^. Patients received lymphodepleting chemotherapy either with bendamustine 90mg/m^2^ for 2 days (8 pts) or with bendamustine 70 mg/m^2^ and fludarabine 30 mg/m^2^ for 3 days (10pts). Patients had a median of 8.5 prior treatments, including BV, checkpoint inhibitors, and auto- and alloSCT. Toxicity was not dose-related and included grade 1–2 cytokine release syndrome (CRS) in three patients. Among 14 patients with evidence of disease prior to lymphodepletion, 6 (43%) achieved CR (all in the cohort benda/flu), 1 (7%) had a PR, 2 (14%) had SD, and 5 (35%) had PD at DL2 [159].

In a phase one study, Wang et al. evaluated the infusion of CD30-targeted CAR-T cells in 17 very heavily pretreated patients with rr-cHL in China, 53% of whom had failed autoSCT and 25% BV. Treatment was well tolerated, with grade ≥3 non-hematologic toxicity occurring in only 1/18 patients. Infusion-related toxicity was restricted to brief, self-limited febrile syndrome appearing 30–120’ after the infusion. The ORR was 35% with 6/17 PRs and six additional patients (35%) with SD. The median PFS was 6 months with 2 ongoing PRs at 12–14 months [160]. 

In another pre-clinical study, it was shown that anti-CD123-CAR-T cells can effectively target both the malignant cells and TAMs, thus overcoming immunosuppression of the tumor microenvironment and potentially eradicating the disease in immunodeficient mice [161]. In addition, Bollard et al. reported the infusion of autologous T-cells against EBV-antigen latent membrane protein 1 and 2 (LMP1 and LMP2) in 25 patients with EBV-associated HL with a ~50% ORR in patients with active, relapsed disease and maintenance of the remission status in all patients who received the product as consolidation [162].

## 6. Conclusions

The novel, highly active immunotherapies, BV and checkpoint inhibitors, have improved the outcome of patients with rr-cHL over the last few years and have prevailed over the development of other, modestly active agents that are typically small-molecule inhibitors. The Checkmate 205 and KEYNOTE-087 phase II trials confirmed the high response rates to nivolumab and pembrolizumab, respectively, which were widely applicable in the pre-specified different clinical circumstances of rr-cHL in terms of BV and ASCT pretreatment, the mid-term durability of responses, as well as the well-tolerated side effects of checkpoint inhibitors. After appropriate testing, sintilimab and tislelizumab might provide additional options for rr-cHL in the context of the rising cost issues, which are becoming increasingly significant. The synergistic effect between checkpoint inhibitors and hypomethylating agents, such as decitabine, is also highly promising. Despite a lack of randomized trials, which precludes formal proof, the clinical feeling is that the OS of patients with rr-cHL after autoSCT failure (or ineligible for autoSCT chemorefractory patients) has been prolonged. In fact, in the majority of cases, the almost universally lethal rr-cHL can be transformed into a chronic, smouldering disease for prolonged time periods. 

Potential improvements in autoSCT eligibility and success rates can probably be achieved in the future by the incorporation of BV or checkpoint inhibitors into the traditional second-line regimens. The need for subsequent therapy after autoSCT, and especially alloSCT, can also be reduced with BV consolidation for one year, while pembrolizumab was also tested in this setting. Combinations of active drugs in chemo-free approaches may further improve the results in terms of efficacy and toxicity. 

Finally, BV is gradually being incorporated into the first-line therapy of cHL with improvements in disease control and potentially OS at least in subgroups of patients with advanced disease, while similar applications of nivolumab and pembrolizumab appear promising.

## Figures and Tables

**Table 1:** Approved indications of brentuximab vedotin, nivolumab, and pembrolizumab according to the European Medicines Agency (EMA) and the US Food and Drug Administration (FDA).

EMA: European Medicines Agency	FDA: US Food and Drug Administration
**Brentuximab vedotin**	
1. rr-cHL, CD30+ following	1. rr-cHL after failure of
1.1 autoSCT or	1.1 autoSCT or
1.2 ≥2 prior therapies, when autoSCT or multi-agent chemotherapy is not a treatment option	1.2 ≥2 prior multi-agent chemotherapy regimens in patients who are not autoSCT candidates
2. CD30+ HL at increased risk of relapse or progression following autoSCT	2. cHL at high risk of relapse or progression, as post autoSCT consolidation
3. Adult patients with previously untreated stage IV, CD30+ cHL, in combination with AVD *	3. Previously untreated stage III/IV cHL, in combination with AVD *
**Nivolumab**	
1. As monotherapy in adult patients with rr-cHL after autoSCT and treatment with BV	1. Adult patients with cHL that have relapsed or progressed after
	1.1 autoSCT and BV or
	1.2 ≥3 lines of systemic therapy that included autoSCT
**Pembrolizumab**	
1. As monotherapy in adult patients with rr-cHL	1. Adult and pediatric patients with refractory cHL, or who have relapsed after ≥3 prior lines of therapy
1.1 who have failed autoSCT and BV, or	
1.2 who are transplant-ineligible and have failed BV	

HL = Hodgkin lymphoma; cHL = classical Hodgkin lymphoma; rr-cHL = relapsed/refractory classical Hodgkin lymphoma; autoSCT = autologous stem cell transplantation; BV = brentuximab vedotin. * AVD = combination of doxorubicin, vinblastine, and dacarbazine.

**Table 2:** Summary of studies of brentuximab vedotin in chemorefractory, transplant-ineligible patients with relapsed/refractory classical Hodgkin lymphoma.

Patients’ Characteristics and BV Efficacy Measures	UK-Wide [68]	Italian [69]	USA [70]	German [71]	Greek [58]	Italian [57]
**Patients (#)**	99	30	15	9	20	71
**Age (median (range))**	32 (13–70)	27 (NR)	31 (16–64)	36 (24–71)	27	24% > 60 y
**ECOG PS ≥ 2**	5%	NR	NR	44%	NR	0%
**Previous lines of Tx (median(range))**	2 (2–4)	2 (2–4)	2 (2–4)	3 (2–6)	3 (2–11)	NR
**Salvage regimen**	P/G	P/G/B	P	NR	NR	NR
**Response to salvage regimen**						
Refractory (SD + PD)	52% (15 + 37)	PET + in all pts	73% (60 + 13)	89%	75%	NR
PR	38%	PET + in all pts	27%	11%	20%	NR
CR	10%		0%		5%	NR
**Time from last treatment to BV (months; median (range))**	2.5 (0.7–34.8)	NR	NR	2 (1–22)	NR	NR
**Response to BV**						
ORR	56%	40%	NR	55%	NR	51%
CR	29%	30%	53%	33%	NR	25%
**Subsequent autoSCT (or allo)**						
Proceeded directly	34% *	47% ^†^	80% ^§^	44%	40% ^¶^	NR
Proceeded after further Tx	27%	13%	20%	0%	0%	NR
No SCT	39% **	40%	0%	55%	60% ^¶^	NR

PS = performance status; Tx = treatment; SD = stable disease; PD = progressive disease; PR = partial remission; CR = complete response; ORR = overall response rate; SCT = alloSCT = allogeneic stem cell transplantation; NR = not reported; P =platinum-based; G = Gemcitabine-based; B = BEACOPP. * Almost all had responded to BV; 2/3 had CR after BV; ** typically refractory to BV and subsequent therapy, if given; 26% achieved short-lived PRs of 3.6 months median duration; ^†^ including 14 patients; nine in CR and five refractory to BV, ^§^ including 12 patients; eight in CR and four refractory to BV; ^¶^ Among 8/20 patients who proceeded to autoSCT only three responded (all PR); among 12/20 who did not undergo SCT, two responded (CR and PR) but refused further therapy.

**Table 3:** Main patient characteristics and outcome measures in the AETHERA trial. Data extracted from references [75,76].

Outcome	Brentuximab Vedotin	Placebo	Hazard Ratio (95% Confidence Intervals)
Two-year PFS per IRF	63%	51%	0.57 (0.40–0.81) **
Two-year PFS per investigator	65%	45%	0.55 (0.39–0.77)
Five-year PFS per investigator	59%	41%	0.52 (0.38–0.72)
≥2 risk factors			0.42 (0.30–0.60)
≥3 risk factors			0.39 (0.26–0.60)
Subsequent anti-neoplastic therapy (five-year report)	32%	54% *	NR (*p* < 0.0001)
Five-year probability of treatment with ≥ 2 subsequent therapies or death	36%	46%	0.66 (0.47–0.92)
Number of alloSCTs (five-year report)	19 (12%)	35 (21%)	NR
Number of deaths (five-year report)	40	37	NR
Number of second malignancies/MDS-AML	6/3	3/2	NR
Peripheral sensory neuropathy (all/≥ grade 3)	56%/10%	16%/1%	NR
Peripheral motor neuropathy (all/≥ grade 3)	23%/6%	2%/1%	NR
Neutropenia (≥ grade 3)	29%	10%	NR

IRF = independent review facility; NR = not reported; MDS-AML = myelodysplastic syndrome, acute myeloid leukemia; * 87% of relapsed patients in the placebo arm received BV (crossover); ** *p* = 0.0013.

**Table 4:** Findings of the phase III ECHELON-1 randomized trial comparing six cycles of BV-AVD versus ABVD in stage III/IV cHL [17,77,78,80,84].

Patients’ Characteristics and Key Outcome and Toxicity Measures	BV–AVD	ABVD	Difference	Hazard Ratio (95%CI)	*p*-Value
**Patients (#) and patient characteristics**	*n* = 664	*n* = 670			
Age (median(range))	35 (18–82)	37 (18–83)		NA	
Stage IV (%)	64	63		NA	
IPS 4–7 (%)	25	27		NA	
**Outcome measures**					
*All patients*					
Two-year mPFS per IRC (%) (primary endpoint)	82.1	77.2	+4.9%	0.77 (0.60–0.98)	0.03
Two-year mPFS per INV (%)	81.0	74.4	+6.6%	0.73 (0.67–0.92)	0.007
Two-year OS (%)	96.6	94.9	+1.7%	0.72 (0.44–1.17)	0.19
*Stage IV or extranodal site subgroup*					
Two-year mPFS per IRC, Stage IV (%)	82.0	74.9	+7.1%	0.71 (0.53–0.96)	0.023
Two-year mPFS per IRC, extranodal ≥1 (%)	82.4	74.9	+7.5%	NR	0.018
Two-year mPFS per IRC, extranodal ≥2 (%)	80.2	71.1	+9.1%	0.67 (0.44–1.00)	0.049
Two-year OS, stage IV (%)	97.4	93.4	+4.0%	0.51 (0.27–0.97)	0.037
Two-year OS, extranodal ≥ 1 (%)	97.5	93.4	+4.1%	0.43 (0.22–0.85)	0.013
*Elderly patients (* *≥ 60 years)*	*n = 84*	*n = 102*			
Two-year mPFS per IRC, all elderly (%)	70.3	71.4	−1.1%	1.00 (0.58–1.72)	0.99
Two-year mPFS per IRC, stage IV elderly (%)	71.3	66.1	+5.2%	0.80 (0.42–1.53)	0.51
Two-year mPFS per INV, stage IV elderly (%)	74.0	59.9	+14.1%	0.66 (0.35–1.27)	0.21
**Toxicity**					
Toxic deaths, all patients [# (%)]	9 (1.4)	13 (1.9)	−0.5%	-	-
Toxic deaths, elderly [# (%)]	2/84 (2.4)	5/102 (4.9)	−2.5%	-	-
Hospitalization, all patients (%)	37	28	+9%	-	-
Peripheral sensory neuropathy, all patients, all grades (%)	29	17	+12%	-	-
Peripheral sensory neuropathy, all patients, grade ≥ 3 (%)	5	< 1	+4%	-	-
Peripheral motor neuropathy, all patients, all grades (%)	11	4	+7%		
Febrile neutropenia, all patients (%)	19	8	+11%	-	-
Febrile neutropenia, elderly (%)	37	17	+20%	-	

ABVD = Adriamycin, Bleomycin, Vinblastine and Dacarbazine; BV-AVD = Brentuximab Vedotin, Adriamycin, Vinblastine and Dacarbazine; IPS = International Prognostic Score.

**Table 5:** Brentuximab vedotin combinations or monotherapy as first-line therapy in patients with advanced or predominantly advanced stage Hodgkin lymphoma (both approved and non-approved indications).

Treatment	Patients (#)	Stage III/IV (%)	Age [Med (Range)]	PS ≥ 2(%)	RT (%)	ORR (CR), %	PFS (at *n*-yrs)	OS (at *n*-yrs)
ABVD × 6 (ECHELON-1) [17]	102	100	66, (60–83)	10	NR	83 (70)	71.4% (2)	17 deaths
BV-AVD × 6 (ECHELON-1) [17]	84	100	68, (60–82)	12	NR	86 (73)	70.3% (2)	15 deaths
BV × 2 + AVD × 6 + BV × 4 [86]	48	81	69, (60–88)	19			84(2)	93(2)
B-CAP [87]	49	95	66, (60–84)	12	21	98 (65)	74(1)	93(1)
BV-DTIC × 12/3 ws [88] *	22 *	72	69, (62–88)	32	5	100 (62)	median 17.9 mo ^§^	nr ^§§^
BV-Bendamustine × 6 [88] *	20 *	75	75, (63–86)	20	12	100(80)	nr ^†^	nr ^†^
BV-Bendamustine × 6 (HALO) [89]	22	82	70, (62–79)	NR	NR	87(87)	5/15 pts	NR
BV alone [90] *	27 *	63	78, (64–92)	22	NR	92 (73)	median 10.5 mo ^††^	median 11.8 mo ^††^
BV alone (BREVITY) [91]	38	82	76, (59–90)	50	NR	84(26)	median 7.4 mo	NR

RT = radiotherapy; PFS = progression-free survival; OS = overall survival; NR = not reported; nr = not reached. * ≥60 years and ineligible or declined ABVD or BEACOPP: Median ejection fraction 60 (25–72), 60 (45–70), and 65 (49–74) for BV–DTIC, BV–Benda, and BV alone. ^§^ ≈49% at 2 years; ^§§^ nr = not reached after a median follow-up of 21.6 months; ^†^ nr = not reached after a median follow-up of 10.8 months, PFS at 18 months ≈ 61%, Treatment-related mortality 10%; ^††^ ~30% 18-month PFS and ~38% 18-month OS (derived from curves).

**Table 6:** Summary of clinical trials combining brentuximab bedotin with salvage regimens (typically used to mobilize stem cells) prior to autoSCT either in sequential or concurrent design.

Author	Regimen	Pts (#)	Median Age (Range)	Primary Refractory (%)	BEACOPP (%)	CR Definition	ORR (%)	CMR (%)	ASCT Performed (%)	P(E)FS
Moskowitz AJ [92,93]	BV × 2 * plus AugICE if no CMR	45	31, (13–65)	56	7	D5PS 1–2	NR	27 to BV, 76 to both **	98 **	80% at 3 years
Moskowitz AJ [93]	BV × 3 * plus AugICE if no CMR	20	35, (19–59)	45	NR	D5PS 1-=2	NR	30 to BV, 80 to both	100	85% at 2 years
Chen R [94] & Herrera A [95]	BV × 2–4 ^§^ plus chemo if no CMR	37	34, (11–67)	65	5	Per Cheson 2007	68 to BV	35 to BV, 75 to both ^§§^	92 ^§§§^	72% at 2 years ^¶¶^
Herrera A [96]	BV × 4 ^¶^ plus additional Tx at phys’s discretion	20	25, (15–57)	60	0	Per Cheson 2007	75 to BV	50 to BV, 70 to both	90, (18/20)	NR
Garcia-Sanz R [97]	BrESHAP × 3 + BV × 1 plus consBV × 3	66	36, (18–66)	61	3	Per Cheson 2007	91	70 ^¶¶¶^	91	71% at 2.5 years
Hagenbeek A [98]	BV–DHAP × 3	61	29, (19–71)	38, (no CR)	18	NR	87	79	87	76% at 2 years
Cassaday RD [99]	BV–ICE × 2 ^†^	16	32, (23–60)	69, (no CR)	0	Per Cheson 2007	94	88	75	19% relapses at medfup 6.5 mo
LaCasce AS [100]	BV–Benda up to 6 plus cons BV up to 16	55	36, (19–79)	51, (no CR)	0	Per Cheson 2007	92	74	75 †	70% at 2 years †
Herrera A [102]	BV–Nivo	62	36, (18–69)	45	3	Per Lugano 2014	83	50 ^††^	89 ^†††^	89% at 6 months

CR = Complete Remission; ORR = Overall Response Rate; CMR = Complete Metabolic Remission; ASCT = autologous stem cell tranaplantation; P(E)FS = Progression (Event) Free Survival. * BV 1.2 mg/kg on days 1, 8, 15 of each cycle; ^§^ Standard BV cycles 1.8 mg/kg every 21 days; ^¶^ Escalated to 2.4 mg/kg every 21 days if no CMR achieved with two standard 21 day cycles at 1.8 mg/kg; ^†^ BV 1.5 mg/kg on days 1 and 8 combined with ICE every 21 days. ** 80% (36/45) if D5PS score 3 considered as CR. A single patient LFU after a positive PET with BV × 2. ^¶¶¶^ 76% if D5PS score 3 considered as CR (similar outcomes for D5PS scores 3 and 2). ^††^ 60% if D5PS score 3 considered as CR. ^§§^ Five additional patients were forwarded to autoSCT directly after BV with a positive PET (4 PR, 1 SD with IF-RT). ^§§§^ Including two patients who received alloSCT for PR and SD after chemo. ^¶¶^ Only the 32/37 patients who received autoSCT were included (80% for those transplanted after BV only). ^†††^ 42 patients proceeded to autoSCT after BV plus Nivo and 12 after additional salvage therapy. † PFS restricted to patients who received autoSCT (most after BV-Benda × 2). Several patients did not undergo autoSCT for willingness or logistic reasons despite being eligible.

**Table 7:** Multicenter phase II trials evaluating the combination of brentuximab vedotin with AVD (sequential or concurrent) with or without radiotherapy on limited stage classical Hodgkin lymphoma.

Study	Intervention	Patients	Age (Median (Range))	Bulk and RF (EF/EU (%))	Metabolic CR Rates	RT (%)	Median Follow-Up	PFS	OS
Abramson JS [110]	BV × 2(d1 + 15) →PET, BV-AVD × 4–6, based on PET	34	36 (20–75)	non-bulky (all) 62/38	BV × 2: 18/34 (50%)	0	14 mo	90%	97%
					BV-AVD × 2: 33/33 * (100%)				
					BV-AVD × 4: 30/32 * (94%)				
Park [109]	ABVD × 2–6 (RF/iPET) **, BV × 6 (six weeks later)	40	29 (19–68)	non-bulky (all) 55/45	ABVD × 2: 29/40 (73%) (D5PS 1–2)	2.5	22 mo	92% at 2yrs	97% at 2yrs
					37/40 (93%) (D5PS 1–3)				
					EOT: 37/39 ** (95%) (D5PS 1–2)				
					38/39 (97%) (D5PS 1–3)				
Kumar [111]	BV-AVD × 4 30 Gy ISRT if PET-neg	30	31 (18–59)	Bulky 57% 0/100 †	BV-AVD × 2: 14/29 (48%) (D5PS 1–2)	83 ^§^	18.8 mo	93% at 1 yr	NR
					26/29 (89%) (D5PS 1–3)				
					BV-AVDx4: 24/29 (82%) (D5PS 1–2)				
					27/29 (92%) (D5PS1–3)				
					EOT: 25/25 ^§§^				

EOT = end-of-treatment, RF = risk factors, EF/EU = early favorable or early unfavorable according to EORTC or German Hodgkin Study Group (GHSG) definition, NR = not reported. † Stage IIBX and/or IIBE included (GHSG definition), * one patient removed due to toxicity (gr.2 hypersensitivity and toxic death after AVD × 1 in an elderly patient, ** 11, 26, and 3 patients (27.5%, 65%, 7.5%, respectively) received 2, 4, or 6 AVD cycles, respectively; † Stage IIBX and/or IIBE included (GHSG definition); One toxic death on BV (sepsis/hepatic failure), ^§^ Five patients did not receive RT: 2 PD, 1 withdrawal after BV-AVD × 1 (gr.3 neuropathy), 2 patient’s desire, ^§§^ including 2 patients with false-positive EOT-PET.

**Table 8:** Comparison of patients’ characteristics and overall results for nivolumab, pembrolizumab, sintilimab, and tislelizumab phase II trials for rr-cHL.

Patients’ Characteristics and Key Outcome and Toxicity Measures	Nivolumab [124,127,128]	Pembrolizumab [125,126]	Sintilimab [133]	Tislelizumab [134]
**Trial Name/Code**	Checkmate 205	KEYNOTE-087	ORIENT-1	BGB-A317-203
**Location**	Europe, North America	Europe, North America, Israel, Australia, Japan	China	China
**Dose/Schedule**	3 mg/kg every 2 weeks	200 mg every 3 weeks	200 mg every 3 weeks	200 mg every 3 weeks
**Duration of treatment**	Until PD or unacceptable toxicity ^§^	Until PD or unacceptable toxicity or investigator decision or max of 24 months ^¶^	Until PD or unacceptable toxicity or max of 24 months	Until PD or unacceptable toxicity
**Treatment beyond progression**	Accepted per early protocol amendment (see text)	Permitted for clinically stable patients if agreed on by investigator and sponsor	Accepted for clinically stable patients if agreed on by investigator and sponsor	NR
**Inclusion criteria**	3 different clinical scenarios (arms A, B, C) always after autoSCT and after BV in Arms B and, partly, C (see Table 9)	3 different clinical scenarios (cohorts 1, 2, 3) after autoSCT (cohorts 1, 3) and after BV (cohorts 1, 2, and partly 3) (see Table 9)	rr-cHL after ≥2 lines of Tx (previous autoSCT and BV not required) in 18 Chinese hospitals	rr-cHL after autoSCT failure or ≥2 lines of Tx if autoSCT ineligible in 11 Chinese hospitals
**Primary endpoint**	ORR by IRC [55]	ORR by IRC [55] and safety	ORR by IRC (PET or CT)	ORR by IRC [129]
**Patients (#)**	243	210	92	70
**Age (median (Range))**	34 (26–46) †	35 (18–76)	33 (28–43)	32.5 (NR)
**Age ≥ 65 years (%)**	6 ^§§^	8.6	0	6
**ECOG PS 0–1 (%)**	100	100	99	NR
**Previous lines of Tx (median (range))**	4 (3–5) †	4 (1–12)	3 (2–5)	3 (2–11)
**≥3 lines of previous Tx (%)**	85	87	68	NR
**Ineligible for autoSCT (%)**	0	39	NR	81 *
**Previous ASCT (%)**	100	61	19	19
**Previous BV (%)**	74	83	6	21 **
**Median follow-up (months)**	33.0	27.6	10.5	7.9
**ORR per IRC (%)**	71	72	80	86
**CR rate per IRC (%)**	21	28	34	61
**Progression free survival (PFS)**	15 mo (median)	13.7 mo (median)	77.6% at 6 months	80% at 6 months
**Duration of response**	18 mo (median) ††	16.5 mo (median) ††	~79% at 6 months	NR
**Overall survival**	~87–88% at 2 yrs	90.9% at 2 yrs	No deaths	1 death of PD
**Discontinuation (patient number (%))**	26 (11%) ^¶^	14 (6.7%) ^¶^	3 (3%) ^¶^	4 (5.7%)
**Toxicity**				
**TRAEs in ≥ 10% of patients**	Rash, fatigue, diarrhea, pruritus, nausea, IRRs	Rash, fatigue, hypothyroidism, pyrexia	Pyrexia, rash, hypothyroidism, pneumonitis, increased ALT, leukopenia	Pyrexia, hypothyroidism, increased weight, upper respiratory tract infection, cough
**TRAEs gr. 3/4 in ≥ 2% of patients**	lipase elevations, neutropenia, ALT elevations	neutropenia	pyrexia, IRRs, lung infection	pneumonitis, upper respiratory tract infection
**TRAEs of special interest**	hypothyroidism/thyroiditis (12%), pneumonitis (4%), hyperthyroidism (2%) but none gr. 3/4, rash 9%, hepatitis 5% (4% gr. 3/4)	hypothyroidism (16%), pneumonitis (5%), hyperthyroidism (4%) but none gr. 3/4	hypothyroidism (20%), pneumonitis (10%; only 1% gr.3/4)	hypothyroidism (30%), pneumonitis

IRC = independent review committee; IRRs = infusion-related reactions; NR = not reported; TRAEs = treatment-related adverse events. NOTE: Comparisons are not meaningful between nivo/pembro and sintilimab/tislelizumab because of highly different eligibility criteria and follow-up times. Even toxicities are difficult to compare due to the very different follow-up times. * 76% due to chemorefractoriness; ** immunotherapy in general, including BV; ^§^ In arm C only, patients were to discontinue nivolumab after one year in persistent CR and treatment could be resumed if relapse occurred within two years from the last dose; ^¶^ Patients attaining CR could stop treatment after a minimum of six months and ≥2 doses after CR; † numbers in parentheses are interquartile range (IQR); ^§§^ ≥60 years; ††median duration of response for CRs versus PRs: for nivolumab 32 versus 13 months and for pembrolizumab not reached versus 10.9 months; ^¶^ most frequent causes; Nivolumab: IMRAEs including pneumonitis (2%) and autoimmune hepatitis (1%); Pembrolizumab: pneumonitis (3%), IRRs (1%), single cases of various IMRAEs; Sintilimab: pneumonitis (*n* = 2 plus thrombocytopenia in 1), liver function abnormalities (*n* = 1); Tislelizumab: pneumonitis (*n* = 2), organizing pneumonia (*n* = 1), focal segmental glomerulosclerosis (*n* = 1).

**Table 9:** Patients’ characteristics and outcomes of Checkmate 205 and KEYNOTE-087 trials of nivolumab and pembrolizumab, respectively, by arm/cohort.

Patients’ Characteristics and Key Outcome and Toxicity Measures	CHECKMATE-205 (Nivolumab) [124,127,128]			KEYNOTE-087 (Pembrolizumab) [125,126]		
	Arm A	Arm B	Arm C	Cohort 1	Cohort 2	Cohort 3
**Description of Arm/Cohort**	Failed autoSCT but no prior BV	Failed autoSCT and subsequent BV	Failed autoSCT but exposed to BV before and/or after autoSCT *	Failed autoSCT and subsequent BV	Ineligible for autoSCT; Failed salvage chemo and BV	Failed autoSCT but no subsequent BV given (may have failed prior BV)
**Patients (number)**	63	80	100	69	81	60
**Median follow up**	33.0 months (all three arms combined)			27.6 months (all three cohorts combined)		
**Age (median; years)**	33	37	32	34	40	32
**PS 0 (%)**	62	53	50	42	54	48
**Previous Tx lines (median)**	2	4	4	4	4	3
**≥3 previous lines of Tx (%)**	46	100	97	99	96	60
**Previous autoSCT (%)**	100	100	100	100	0	100
**Previous BV (%)**	0	100	100	100	100	42
**ORR per IRC**	65	71	75	77	67	73
**CR rate per IRC**	32	14	20	26	26	32
**Progression free survival (median; months)**	17	12	15	77	67	73
**Duration of response (median; months)**	25 **	16 **	18 **	22.1 †	11.1 †	24.4 †
**Overall survival at two-years (%)**	90	86	86	92.5	90.6	89.4

* In arm C patients were to discontinue nivolumab after one year in persistent CR and treatment could be resumed if relapse occurred within two years from the last dose; ** Data presented at ASH 2018; not precisely reported in ref [128]; † For CRs versus PRs: Cohort 1 25 versus 19.5 months, cohort 2 19.2 versus 7.9 months, cohort 3 not reached versus 13.9 months.

**Table 10:** Outcomes and toxicity of alloSCT after exposure to checkpoint inhibitors.

Characteristics/Outcomes/Toxicities	Merryman et al. [142]	Armand et al. (Checkmate 205) [124]
Patients (number)	31	44
Time from last PD1 dose to alloSCT (days; median (range))	62 (7–260) *	49 (IQR 31–127)
Salvage between PD1 and alloSCT (%)	49 *	27
Hyperacute GvHD (within 14 days) (%)	NR	11
Cumulative Incidence of aGvHD (grade 2–4) at 6 mo/1 yr	NR/45	30/45 **
Cumulative Incidence of aGvHD (grade 3–4) at 6 mo/1 yr	NR/26	20/32 **
Cumulative Incidence of cGvHD at 6 mo/1 yr	NR/33	15/20 **
Liver Sinusoidal Obstructive Syndrome	6% at 3 months	2%
Non-infectious Febrile Syndrome (%)	18 *	9
Other Toxicities	-	Encephalitis 2%
Treatment-Related Deaths (number, %)	3 (10%)	5 (11%)
Cumulative Incidence of Non Relapse Mortality at 6 mo/1 yr (%)	NR/10	13/13 **
Cumulative Incidence of Relapse at 6 mo/1 yr (%)	NR/16	7/13
Progression Free Survival at 6 mo/1 yr (%)	NR/74	82/77
Overall Survival at 6 mo/1 yr (%)	NR/90	87/79

aGvHD = acute graft versus host disease; NR = not reported. * among the total of 39 patients included in the study (8 with non-Hodgkin’s lymphomas); ** one-year rates approximated from the published survival curves.
